# An Epidemiologic Approach for Estimating Risk Reduction and Asymptotic Power on the Log-Difference Scale

**DOI:** 10.3390/ijerph22050719

**Published:** 2025-05-01

**Authors:** Jimmy T. Efird

**Affiliations:** 1 Cooperative Studies Program Coordinating Center, VA Boston Healthcare System, Lafayette City Center, 2 Avenue de Lafayette, Boston, MA 02111, USA; jimmy.efird@va.gov or jte38@case.edu; Tel.: +1-650-248-8282; 2 Department of Radiation Oncology, School of Medicine, Case Western Reserve University, Cleveland, OH 44206, USA

**Keywords:** clinical trials, common referent-control, conditional independence, multiplicity adjustment, power, risk reduction, sample size

## Abstract

When comparing the efficacy or harmfulness of two groups (e.g., drugs, devices, assays, interventions, environmental toxins), it is important to minimize bias by making this comparison with respect to a common referent-control group, assuming random allocation. Under such a scenario, one can estimate risk reduction for a new therapy on a log-difference, relative effect scale. The current manuscript reviews the large-sample framework for this conditionally independent comparison and demonstrates how to estimate test power for a given sample size.

## 1. Introduction

A frequently used clinical trial design is to directly compare a new treatment against a standard therapeutic compound. However, many years may have passed since the standard compound was originally approved. During this time, variations in the disease process, manufacturing changes, or practice deviations may have occurred, such that the standard treatment is no longer efficacious, or is effective to a much lesser degree than originally marketed.

Observing a statistically significant absolute effect (risk) reduction (i.e., the standard minus new therapy event rate) may not necessarily prove the clinical usefulness of the new treatment unless the clinical trial design includes a common comparison group as a referent-control. This contrast allows for the side-by-side evaluation of the new and standard agents in the context of relative effect reduction on the log-difference scale. Importantly, the use of a shared referent-control arm minimizes bias and threats to internal validity compared with assessing the absolute risk difference.

Power and sample size are important components in the planning of a clinical trial. However, the literature is largely silent on the topic of a conditionally independent shared comparison arm when assessing risk reduction on the log-difference, relative effect scale.

## 2. Preliminaries

### 2.1. Conditionally Independent, Large-Sample Distribution

Consider a test to compare two relative effect estimates (REEs), denoted as $\left({\hat{\vartheta }}_{1},\;{\hat{\vartheta }}_{2}\right),$ on the log-difference scale, with respect to a common referent-control group. Approximate log-normally distributed REEs include relative risks (RR), odds ratios (OR), and hazard ratios (HR). A variance-stabilizing, logarithmic transformation tends to make the difference of REEs closer to a normal distribution by reducing any potential relationship between the variance and mean. The application of this transformation is especially useful when dealing with probabilities near 0 or 1.

Assuming that (a) the standard error (SE) for the logarithm of $\left({\hat{\vartheta }}_{1}\right)$ does not differ considerably from that for $\left({\hat{\vartheta }}_{2}\right)$, (b) corresponding sample sizes $\left(n_{{\hat{\vartheta }}_{1}},n_{{\hat{\vartheta }}_{2}}\right)$ are reasonably large, (c) ${\hat{\vartheta }}_{1}$ and ${\hat{\vartheta }}_{2}$ with a common referent-control arm are asymptotically consistent, but not necessarily unbiased, estimates of the true parameters, and (d) the large-sample distribution for the logarithm of $\;\left({\hat{\vartheta }}_{1},{\hat{\vartheta }}_{2}\right)$ is approximately Gaussian, with estimated event rates for both groups not being too close to 0 or 1, it follows from the Le Cam–Lévy–Ferguson “Conditional Martingale Limit Theorem” that we obtain the following equation:

(1) $$\underset{\left(n_{{\hat{\vartheta }}_{1}},n_{{\hat{\vartheta }}_{2}}\right)\to \infty }{\lim}\frac{log\left(\frac{{\hat{\vartheta }}_{1}}{{\hat{\vartheta }}_{2}}\right)-E\left[log\left(\frac{{\hat{\vartheta }}_{1}}{{\hat{\vartheta }}_{2}}\right)\right]}{\sqrt{Var\left[log\left(\frac{{\hat{\vartheta }}_{1}}{{\hat{\vartheta }}_{2}}\right)\right]}}\sim \;N(0,1),$$

where $E\left[*\right]$ represents the expectation, and N(0,1) is the standard normal distribution [1]. The single tilde, “~”, indicates asymptotic equivalence of the same magnitude, under the assumption that the underlying Martingale processes are stationary, ergodic, and F-measurable [2], with finite variance.

The cumulative distribution function for $log\left(\frac{{\hat{\vartheta }}_{1}}{{\hat{\vartheta }}_{2}}\right)$ divided by that for a standard normal curve approaches unity for large sample sizes. Under asymptotic regularity conditions, ${\hat{\vartheta }}_{1}\;$ is conditionally independent of ${\hat{\vartheta }}_{2}$, given a common referent-control group. That is, the probability (*A*) for an observation in the numerator of ${\hat{\vartheta }}_{1}\;$ is not affected by the probability (*B*) for an observation in the numerator of ${\hat{\vartheta }}_{2},$ given the probability (*C*) for a shared observation in the denominators of ${\hat{\vartheta }}_{1}\;and\;{\hat{\vartheta }}_{2}.$ Mathematically stated, it follows that (A ⫫$\;B\;\left|\;C\right)\;$ is equivalent to (B ⫫$\;A\;\left|\;C\right).$

The denominator of the limit described above, denoted as $\left(\zeta \right),$ yields the square root of the sample variance estimate of $\left[log\left({\hat{\vartheta }}_{1}\right)-log\left({\hat{\vartheta }}_{2}\right)\right]\;,$ which may be approximated as follows [3]:

(2) $$\zeta \approx \sqrt{{\left\{SE\left[log\left({\hat{\vartheta }}_{1}\right)\right]\right\}}^{2}+{\left\{SE\left[log\left({\hat{\vartheta }}_{2}\right)\right]\right\}}^{2}.}$$

### 2.2. Confidence Interval Method for Estimating the Sample Variance

Lower and upper α–level confidence intervals (CIs) for the indicated REEs are given as $LCI\left[*\right]$ and $UCI\left[*\right]$. That is, for $\left(i=1,2\right),$ we obtain the following expression:

(3) $${CI}_{\alpha }\left(\vartheta_{i}\right)=e^{log\left({\hat{\vartheta }}_{i}\right)\pm z_{\left(\alpha /2\right)}SE\left[log\left({\hat{\vartheta }}_{i}\right)\right]},$$

where $\left(\alpha \right)$ refers to the probability of rejecting the null hypothesis when it is true, and $z_{\left(\alpha /2\right)}$ corresponds to the $100\left(1-\alpha /2\right)$ percentile of $N\left(0,1\right)$ [e.g., $z_{\left(0.05/2\right)}=1.96$]. Using the above bounds and rearranging, it readily follows that we obtain the following equation:

(4) $$\;\;\;\zeta \approx \sqrt{{\left\{\frac{log\left[UCI\left({\hat{\vartheta }}_{1}\right)\right]-log\left[LCI\left({\hat{\vartheta }}_{1}\right)\right]}{2z_{\left(\frac{\alpha }{2}\right)}}\right\}}^{2}+{\;\left\{\frac{log\left[UCI\left({\hat{\vartheta }}_{2}\right)\right]-log\left[LCI\left({\hat{\vartheta }}_{2}\right)\right]}{2z_{\left(\frac{\alpha }{2}\right)}}\right\}}^{2}}.$$

### 2.3. Alternative Computational Formula for $SE\left[log\hat{\left(RR\right)}\right]$

Let $\left(a_{i,j}\right)$ denote the $\left({i^{th},j}^{th}\right)$ cell sizes for a contingency table with corresponding probabilities of $\left(\pi_{i,j}\right)$. Here, the row exposures and column outcomes are indexed by $\left(i\right)$ and $\left(j\right).$ The relative risk estimate $\left(\hat{RR}\right),$ also known as the risk ratio, is defined as follows:

(5) RR^=a11a1+a21a2+,

where $\left(a_{1,1}\right)$ and $\left(a_{2,1}\right)$ follow a binomial distribution. By applying the delta method (Taylor series approximation for large sample sizes), we obtain the following equations:

(6) $$\;\;\;\;\;\;Var\left\{log\left(\;\hat{RR}\right)\right\}=Var\left\{log\left(\;\frac{a_{11}}{a_{1+}}\right)\right\}+Var\left\{log\left(\;\frac{a_{21}}{a_{2+}}\right)\right\}$$

(7) $$\;\;\;\;\;\approx {\left(\frac{a_{1+}}{a_{11}}\right)}^{2}\left\{\frac{\frac{a_{11}}{a_{1+}}\left(1-\frac{a_{11}}{a_{1+}}\right)}{a_{1+}}\right\}+{\left(\frac{a_{2+}}{a_{21}}\right)}^{2}\left\{\frac{\frac{a_{21}}{a_{2+}}\left(1-\frac{a_{21}}{a_{2+}}\right)}{a_{2+}}\right\}$$

(8) $$\;\;\;\;\;\approx \frac{1}{a_{11}}-\frac{1}{a_{1+}}+\frac{1}{a_{21}}-\frac{1}{a_{2+}}.$$

Accordingly, we obtain the following equation:

$$SE\left[log\left(\hat{RR}\right)\right]\;\approx \sqrt{\frac{1}{a_{11}}-\frac{1}{a_{1+}}+\frac{1}{a_{21}}-\frac{1}{a_{2+}}}.$$

The advantage of this direct formula is the increased computational speed gained by foregoing the CI method for estimating $SE\left[log\left(\hat{RR}\right)\right]$. In the case of ORs and HRs, one can derive similar computational approximations using the delta method.

### 2.4. Percentage Relative Effect (Risk) Reduction

When $\left({\hat{\vartheta }}_{1}>{\hat{\vartheta }}_{2}\right)$, the percentage relative effect reduction $\left(\%RLD\right)\;$ is computed as $100\left(\frac{{\hat{\vartheta }}_{1}}{{\hat{\vartheta }}_{2}}-1\right),$ and vice versa when $\left({\hat{\vartheta }}_{2}>{\hat{\vartheta }}_{1}\right),\;$ i.e., $100\left(\frac{{\hat{\vartheta }}_{2}}{{\hat{\vartheta }}_{1}}-1\right).$ If $\left({\hat{\vartheta }}_{1}={\hat{\vartheta }}_{2}\right)$, a null result is obtained for the $\left(\%RLD\right).$ For example, if $\left({\hat{\vartheta }}_{1}=0.80\right)$ and $\left({\hat{\vartheta }}_{2}=0.60\right)$, then $\left(\%RLD\right)\;$ = $100\left(\frac{{\hat{\vartheta }}_{1}}{{\hat{\vartheta }}_{2}}-1\right)=100\left(\frac{0.80}{0.60}-1\right)=100\left(1.33-1\right)=33$.

### 2.5. Null Hypothesis and p-Value

In the case of a null hypothesis (H_0_),$\;log\left({\hat{\vartheta }}_{1}\right)\;and\;log\left({\hat{\vartheta }}_{2}\right)$ are deemed to be equal, versus the alterative scenario in which they differ [3]. That is, H_0_: $log\left(\frac{{\hat{\vartheta }}_{1}}{{\hat{\vartheta }}_{2}}\right)=0,$ which is equivalent to a null %RLD, compared with H_1_: $log\left(\frac{{\hat{\vartheta }}_{1}}{{\hat{\vartheta }}_{2}}\right)\neq 0.$ In the case of (H_0_), both $log\left(\frac{{\hat{\vartheta }}_{1}}{{\hat{\vartheta }}_{2}}\right)\;and$ $E\left[log\left(\frac{{\hat{\vartheta }}_{1}}{{\hat{\vartheta }}_{2}}\right)\right]$ equal zero.

A *p*-value for the log-difference of two REEs is estimated as follows:

(9) $$P\sim 2\cdot \left\{1-\Phi \left(z\right)\right\},$$

where

(10) Φz=∫−∞ze−x222·πdx

and

(11) $$z\sim \frac{\;log\left({\hat{\vartheta }}_{1}\right)-log\left({\hat{\vartheta }}_{2}\right)\;}{\zeta }.$$

### 2.6. Confidence Interval Decision Criteria

The disjoint CI percentage corresponding to an equivalent α-level test is computed as follows:

(12) $$\left[1-2\Phi \left(-Z_{\left(\alpha /2\right)}\frac{\sqrt{2}}{2}\right)\right]x100\%.$$

This yields slightly wider regions to compensate for the pooled (versus non-pooled) sample variance [4]. For example, given that α = 0.01, 0.05, and 0.10, the difference between the estimates $log\left({\hat{\vartheta }}_{1}\right)\;and\;log\left({\hat{\vartheta }}_{2}\right)$ is declared as statistically significant if the 93.1452%, 83.4224%, and 75.5206% CIs for $\left({\hat{\vartheta }}_{1},{\hat{\vartheta }}_{2}\right)$ do not overlap.

Analogously, the difference between the estimates $log\left({\hat{\vartheta }}_{1}\right)\;and\;log\left({\hat{\vartheta }}_{2}\right)$ may be declared as statistically significant when the following equation excludes unity:

(13) $${CI}_{\alpha }\left(\frac{\vartheta_{1}}{\vartheta_{2}}\right)=e^{log\left(\frac{{\hat{\vartheta }}_{1}}{{\hat{\vartheta }}_{2}}\right)\pm \left\{z_{\left(\alpha /2\right)}\right\}\zeta }$$

## 3. Power and Sample Size

### 3.1. Power

The power of a study indicates how frequently a statistical test will detect the falsehood of an underlying null hypothesis when it is false (i.e., $\mathrm{Pr}\left(reject\;H_{0}|H_{0}\;false\right))$. Let $\left(\beta \right)$ denote the probability of failing to reject the null the hypothesis when it is false (i.e., $\mathrm{Pr}\left(accept\;H_{0}|H_{0}\;false\right)$). It follows that we obtain the following equation:

(14) $$z_{\left(\beta \right)}\;\sim \left\{\left[\frac{log\left(RLD+1\right)}{\zeta }\right]-z_{\left(\alpha /2\right)}\right\}.$$

The area to the left of $z_{\left(\beta \right)}$ under a standard normal distribution yields the desired power at the α-level of statistical significance for a two-sided test. When use of a one-tailed test is desired, $z_{\left(\alpha \right)}$, rather than $z_{\left(\alpha /2\right)}$, is used in this formula.

### 3.2. Sample Size and Variability

The confidence interval method may be used to approximate the standard error for the logarithm of the respective relative effect estimates (${\hat{\vartheta }}_{1},\;{\hat{\vartheta }}_{2}$) corresponding to an initial rxc (row by column) = (n) contingency table, where (n) denotes the total sample size (i.e., combined rows). Cell frequencies of the pilot sample table are increased multiplicatively (in an iterative fashion) to obtain the corresponding standard errors for different sample sizes and corresponding power.

Using this method, it is important that the initial 3 × 2 contingency table be sufficiently large to accurately estimate the sample variability within a specified fraction of the population variance [5]. As a rule of thumb, the proportional width of the α-level CI for the population variance of a continuous variable is defined as follows:

(15) n−11χ1−α2 ,n−12 −1χα2,n−12 ,

This value should be as large as the sample variance, where χα2,n−1 2  is the $100\left(1-\alpha /2\right)$ percentile of the chi-squared distribution with $\left(n-1\right)$ degrees of freedom. When α = 0.05, this corresponds to a minimum sample size of 38. However, in practice, a more conservative sample size of 90 (30 per treatment row) is typically chosen as the starting point to account for the binary outcomes.

### 3.3. Simulation of Observations from a Multinomial Distribution

Given (n) distinct trials, the probability that a mutually exclusive set of (k) non-negative random variates ($A_{1},A_{2},\ldots ,A_{k}$) takes on a particular value ($a_{1},a_{2},\ldots ,a_{k}$) is given as follows:

(16) $$P_{n}\left(A_{1}=a_{1},\;A_{2}=a_{2},\;\ldots ,A_{k}=a_{k}\right)=\frac{n!}{\prod_{i=1}^{k}a_{i}}\;\prod_{i=1}^{k}\pi_{i}^{a_{i}},$$

where

(17) $$P_{n}\left(A_{i}=a_{i}\right)=\pi_{i},\;\sum_{i=1}^{k}\pi_{i}=1,\;\mathrm{and}\;\sum_{i=1}^{k}a_{i}=n.$$

This probability is known as a multinomial distribution because of the following expression:

(18) $$\frac{n!}{\prod_{i=1}^{k}a_{i}}\;\prod_{i=1}^{k}\pi_{i}^{a_{i}}={\left(a_{1}+a_{2}+\ldots +a_{k}\right)}^{n},$$

where the latter is a multinomial series.

The multinomial distribution, as illustrated in Example 2, plays an important role in validating the asymptotic normality of the conditional test statistic.

## 4. Computational Methods

Analyses were performed and validated in SAS 9.4 (Cary, NC).

## 5. Example 1—Comparison of Potassium and Sodium Salts

Potassium salt substitutes can be helpful for lowering sodium intake and controlling high blood pressure. Among a cohort of borderline hypertensive (but otherwise healthy) patients, a team of clinical epidemiologists aimed to determine if randomization to a potassium salt substitute (1500 mg/d) reduces the 24-month risk of major adverse cardiovascular outcomes and non-cancer death compared with a formulary of sodium salt (1500 mg/d), with a common referent-control arm of 2300 mg/d sodium salt.

In a 1:1:1 pilot clinical trial, a 9.52% relative effect (risk) reduction on the log-difference scale was observed at 24 months post-randomization (Table 1). Based on this promising result, the team decided to conduct a larger population-based phase III clinical trial. To achieve at least 80% power for a similar %RLD (discounting dropouts), the new pivotal trial would need to randomize 36,835 patients per study arm at the α = 0.05 level of statistical significance (Table 2; see Appendix A for SAS code).

## 6. Example 2—Monte Carlo Simulation of the Conditional Martingale Limit Theorem 

While a theoretical basis exists for the asymptotic normality and conditional independence of the test statistic [1,2], Monte Carlo methods can be used to validate the large- sample properties of this method.

**Figure 1:**
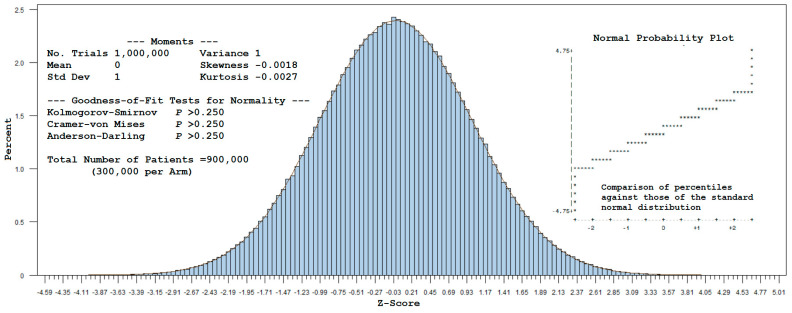
Histogram with a standard normal overlay for simulated z-scores, conditional on a common reference-control arm.

A total of 1,000,000 trials, repeated for each sample of 900,000 patients (300,000 per arm), were drawn from a multinomial distribution using the cell probabilities given in Example 1 (with *T_0_* denoting the common referent-control group). A standardized value was computed for each simulated observation for $log\left(\frac{{\hat{\vartheta }}_{1}}{{\hat{\vartheta }}_{2}}\right)$ by subtracting $E\left(log\left(\frac{{\hat{\vartheta }}_{1}}{{\hat{\vartheta }}_{2}}\right)\right)$ from this estimate and dividing it by $\sqrt{Var\left[log\left(\frac{{\hat{\vartheta }}_{1}}{{\hat{\vartheta }}_{2}}\right)\right]}$. Plotting these values as a histogram yields Figure 1. The corresponding statistics (mean = 0, standard deviation = 1, and approximate zero values for skewness and kurtosis) indicate that the z-scores follow a standard normal distribution, as visually confirmed by the Normal (0,1) curve overlay. Furthermore, normality was not rejected by the Kolmogorov–Smirnov, Cramer–von Mises, or Anderson–Darling tests (see Appendix B for SAS code).

For large samples, this example supports the use of normal-theory methods to estimate power for a 1:1:1 randomized design with a common referent-control arm. In effect, the covariance of $log\left({\hat{\vartheta }}_{1}\right)\;and\;log\left({\hat{\vartheta }}_{2}\right)$ can be disregarded as the sample size increases toward infinity, allowing one to assume an independent, identically distributed (i.i.d.) standard normal test statistic.

## 7. Discussion

### 7.1. Overview

The comparison of two drugs with respect to a common comparator (referent-control) group is a powerful and efficient approach for conducting a clinical trial. Importantly, this method preserves the randomization of the originally assigned patient groups [6]. Additionally, it allows for determining risk reduction on the log-difference scale. The current manuscript presents a novel, computer-based technique for estimating the power of the latter by multiplicatively increasing the cell sizes of an initial pilot sample. This yields the inflated variances needed to compute power for larger sample sizes while still allowing the use of traditional normal-theory methods. As reasoned below, the common referent-control design does not require multiplicity adjustment.

### 7.2. Multiplicity Adjustment

The family-wise type I error rate, which is the probability of at least one false positive test among all hypotheses being tested, is not increased with respect to a common referent-control group [7]. The associated false positive comparison-wise error rate at the individual level is also not inflated, as the “expected proportion of incorrectly rejected hypotheses will not exceed the significance level used in the individual tests” [7,8].

While a treatment is the “family” over which one typically judges the need for multiplicity, there is indistinctness regarding how this entity is defined. Multiplicity control is not required if the underlying therapies have distinct mechanisms of action [9]. That is, “if each decision to reject each individual null hypothesis depends on no more than one significance test, then none of the individual tests constitute a family”, such that an α-level adjustment for any single hypothesis is unnecessary (except if one performs “disjunction testing of a joint intersection null hypothesis”) [10]. While cases exist wherein “the decision on the omnibus null will seem to contradict decisions on individual nulls”, it is important to remember that “rejecting or not rejecting a null is not certain proof that the null is false or true” [10]. Therefore, multiplicity correction for CIs) is needed when the omnibus null can only be evaluated through “a set of surrogate nulls” and cannot be “generalized to testing any diversity of unconnected nulls over the course of a study” [11].

Adjustment is counterintuitive in the case of conjunctive (intersection–union) testing, in which all tests must be significant for the joint null hypothesis to be rejected [9]. However, multiplicity adjustment for CIs is advisable if the comparative therapies are related, as is the case when evaluating “different dosages or regimens of a treatment compared with the same control arm” [12]. This includes trials with collective conditions “if there are no less than two primary hypotheses, unless one assumes that there is an explicit hierarchy in the multiple hypotheses” [12].

Multiplicity adjustment of CIs in the denominator of a relative-effects (log-difference) test of two compounds is generally not performed in practice, as this is equivalent to a stand-alone interaction test with a pooled variance structure [3]. If requested by a regulatory agency, the intervals can be easily adjusted for multiplicity using the Hochberg step-up procedure [13]. However, when “such adjustments are applied unnecessarily, potentially effective treatments may be discarded prematurely” [9].

### 7.3. Limitations

A limitation of REEs is that they are ratios and tend to have a slightly skewed log-normal distribution, which creates issues when the parameters of a test statistic are estimated from the sample. According to Jensen’s inequality, the expectation will be less than or equal to its true value. To some degree, this bias is an advantage because it helps to offset the Cauchy infinite variance problem that may result from taking the ratio of two normally distributed variables [1]. Barring extreme degenerate examples, it is noted that $log\left(\frac{{\hat{\vartheta }}_{1}}{{\hat{\vartheta }}_{2}}\right)$ asymptotically assumes a moderately well-behaved, normal shape in the limit.

The literature offers little consistent advice regarding the lower limit for the sample size needed to satisfy the underlying large-sample assumptions of the test statistic. It is also unclear what level of inaccuracy is incurred when estimating a z-score test statistic when the sample size is small and the observations are not fully independent. By treating the logarithmic transformation of $\frac{{\hat{\vartheta }}_{1}}{{\hat{\vartheta }}_{2}}$ as a mean difference and applying Gauss’ hypergeometric series, one may heuristically conclude that the small-sample test statistic follows a pseudo Behrens–Fisher distribution with $\left(\upsilon \right)$ degrees of freedom [14], as follows:

(19) $$\upsilon =\frac{{\left[\frac{SD\left[log\left({\hat{\vartheta }}_{1}\right)\right]}{n_{{\hat{\vartheta }}_{1}}}+\frac{SD\left[log\left({\hat{\vartheta }}_{2}\right)\right]}{n_{{\hat{\vartheta }}_{2}}}\right]}^{2}}{\left[\frac{{\left\{SD\left[log\left({\hat{\vartheta }}_{1}\right)\right]\right\}}^{2}}{n_{{\hat{\vartheta }}_{1}}+1}+\frac{{\left\{SD\left[log\left({\hat{\vartheta }}_{2}\right)\right]\right\}}^{2}}{n_{{\hat{\vartheta }}_{2}}+1}\right]}-2.$$

An alternative, albeit lower-power, approach is the Lepage test, which treats the problem as a nonparametric test for central tendency and dispersion [15]. In certain situations, nonparametric methods are better suited (less sensitive) for handling violations of independence.

Addressing the exact convergence rates for a conditional Martingale-type central limit theorem, which are ostensibly slower than 1n, is a complex topic beyond the scope of the current manuscript and is best deferred to future research [16]. Nonetheless, it is common practice in many applied scenarios to accept that the underlying data are “close enough” to normal to move forward with normal-theory methods.

Imprecise rounding is also a concern. As REEs are expressed on a logarithmic scale, with significant departures from linearity occurring near unity, it is best to avoid intermediate rounding when computing the test statistic. Fortunately, this rarely poses a problem when the operations are performed by a computer rather than by manual means.

In theory, power for a given sample size may be estimated by simulating datasets from a conditional multinomial distribution, with prescribed cell probabilities being reflective of the underlying hypothesized parameters. Power is then defined as the proportion of the simulated test statistics that fall within the “a priori” alpha rejection region. However, the simulation method is computationally intensive and introduces uncertainty in the values at the distribution tails. As large sample sizes are often needed to detect small effect sizes for risk reduction, the process may be resource-prohibitive or prone to computational errors, in which case normal-theory methods may be the better choice for computing power. While robust estimation methods are available in most standard statistical packages to simulate the covariance of REEs and corresponding test statistics involving correlated data, boundary issues regularly result in algorithmic non-convergence. The simulation of power also is problematic for heavy-tailed, Cauchy-like distributions, which may require sample sizes on the order of millions to properly characterize outlying values. Furthermore, multinomial-type power computations are predicated on the observed pilot sample effect size. Thus, simulation cannot be used to determine power for a range of minimally detectable RLD(%) values based on a single effect size estimate. In such cases, separate pilot samples will be needed to determine the desired range of values. 

### 7.4. Future Directions

Future investigations are directed at estimating power when sample sizes are small and the assumption of conditional independence is questionable. This will include cases where it is difficult to establish the consistency, stability, and normality of underlying data estimates and parameters. Innovative research efforts focusing on nonparametric, two-sample conditional tests and Bayesian methods also may be useful [17,18].

## 8. Conclusions

The use of a common reference-control arm is an important tool for minimizing bias in a randomized clinical trial. Assuming conditional independence, a large-sample method is presented to estimate power for risk reduction on the log-difference scale. This computationally straightforward approach offsets the uncertainty and potential bias associated with tests of absolute risk reduction.

## Figures and Tables

**Table 1:** Pilot analysis (N = 500 participants per arm).

Treatment Regimen (T)		MACE Outcome at 24 Months Post-Baseline *n* (%)		Results ^†^
		**Yes**	**No**	
$T_{0}$	2300 mg/d ^§^ Sodium Salt	50 (10)	450 (90)	${\hat{\vartheta }}_{1}\left(T_{0}:\;T_{2}\right)=1.1905\;\left({CI}_{0.05}=0.8052-1.7602\right)$ ${\hat{\vartheta }}_{2}\left(T_{0}:\;T_{1}\right)=1.0870\;({CI}_{0.05}=0.7428-1.5906)$ $\frac{{\hat{\vartheta }}_{1}}{{\hat{\vartheta }}_{2}}=1.0952\;\left({CI}_{0.05}=0.6346-1.8903\right)$ %RLD=9.52%; P = 0.74395
$T_{1}$	1500 mg/d Sodium Salt	46 (9)	454 (91)	
$T_{2}$	1500 mg/d Potassium Salt	42 (8)	458 (92)	

^†^ Estimates are depicted as rounded values. ^§^ Common referent-control. Baseline = initiation of treatment regimen. ${CI}_{0.05}=$ 95% confidence interval. MACE = major cardiovascular event. mg/d = milligrams per day. *n* = cell frequency. *P* = *p*-value for risk reduction. ${\hat{\vartheta }}_{i}\;$= risk ratio for group (*i*). %*RLD* = percentage relative effect (risk) reduction on the log-difference scale.

**Table 2:** Power analysis for a planned population-based phase III clinical trial.

Sample Size Per Arm	Comparison	${CI}_{0.05}\left(\hat{\vartheta }\right)$ ^†^	$SE\left[log\left(\hat{\vartheta }\right)\right]$ ^†^	Minimal Detectable *RLD* (%)	Power †,§ (%)
10,000	$T_{0}:\;T_{2}$	1.0908–1.2993	0.044615	14	55.7
				12	44.4
	$T_{0}:\;T_{1}$	.9982–1.1836	0.043439	10	33.4
				8	23.5
20,000	$T_{0}:\;T_{2}$	1.1191–1.2664	0.031547	14	84.5
				12	73.0
	$T_{0}:\;T_{1}$	1.0234–1.1544	0.030716	10	58.1
				8	41.6
30,000	$T_{0}:\;T_{2}$	1.1319–1.2521	0.025758	14	95.4
				12	88.3
	$T_{0}:\;T_{1}$	1.0348–1.1417	0.025080	10	75.5
				8	57.2
40,000	$T_{0}:\;T_{2}$	1.1395–1.2437	0.022307	14	98.8
				12	95.4
	$T_{0}:\;T_{1}$	1.0417–1.1342	0.021720	10	86.5
				8	69.6

^†^ Estimates depicted as rounded values. ^§^ Power for a 2-sided, α=0.05 level test. ${CI}_{0.05}=95\%\;confidence\;interval.\;RLD$ = Relative effect (risk) reduction on the log-difference scale $.\;\hat{\vartheta }=Risk\;ratio.\;SE=$ Standard error.

## Data Availability

Data are contained within the article.

## Abbreviations

The following abbreviations are used in this manuscript:

ASCVD	Atherosclerotic cardiovascular disease
CI	Confidence interval
LCI	Lower confidence interval
Log	Logarithm
Mg/day	Milligrams per day
*P*	*p*-value
REE	Relative effect estimate
RLD	Relative effect reduction on the log-difference scale
SD	Standard deviation
SE	Standard error
UCI	Upper confidence interval

## References

[1] Le Cam L. (1986). Asymptotic Methods in Statistical Decision Theory.

[2] Peligrad M. (2011). Conditional central limit theorem via Martingale approximation. arXiv.

[3] Altman D., Bland J. (2003). Interaction revisited: The difference between two estimates. Br. Med. J..

[4] Knol M., Pestmen W., Grobbee D. (2011). The (mis)use of overlap of confidence intervals to assess effect modification. Eur. J. Epidemiol..

[5] Teare M., Dimairo M., Shephard N., Hayman A., Whitehead A., Walters J. (2014). Sample size requirements to estimate key design parameters from external pilot randomized controlled trials: A simulation study. Trials.

[6] Kim H., Gurrin L., Ademi Z., Liew D. (2013). Overview of methods for comparing the efficacies of drugs in absence of head-to-head clinical trial data. B.J.C.P.

[7] Parker R., Weir C. (2020). Non-adjustment for multiple testing in multi-arm trials of distinct treatments: Rationale and justification. Clin. Trials.

[8] Bender R., Lange S. (2001). Adjusting for multiple testing—When and how?. J. Clin. Epidemiol..

[9] Molloy S., White I., Nunn A., Hayes R., Wang D., Harrison S. (2022). Multiplicity adjustments in parallel-group multi-arm trials sharing a control group: Clear guidance is needed. Contemp. Clin. Trials.

[10] Rubin M. (2021). When to adjust alpha during multiple testing: A consideration of disjunction, conjunction, and individual testing. Synthese.

[11] García-Pérez A. (2023). Use and misuse of correction for multiple testing. Meth. Psych. B.

[12] Li G., Taljaard M., Van den Heuvel E., Levine M., Cook D., Wells G., Deveraux P., Thabane L. (2017). An introduction to multiplicity issues in clinical trials: The what, why, when and how. Int. J. Epidemiol..

[13] Hochberg Y. (1988). A sharper Bonferroni procedure for multiple tests of significance. Biometrika.

[14] Kabe D. (1996). On the exact distribution of the Fisher-Behren’s-Welch statistic. Metrika.

[15] Lepage Y. (1971). A combination Wilcoxon’s and Ansari-Bradley’s statistics. Biometrika.

[16] Bolthauser E. (1982). Exact convergence rates in some Martingale central limit theorems. Ann. Prob..

[17] Lee S., Cha S., Kim I. (2024). General framework for conditional two-sample testing. arXiv.

[18] Yan J., Li Z., Zhang X. (2024). Distance and kernel measures for global and local two-sample conditional distribution testing. arXiv.

